# Acoustophoretic Control of Microparticle Transport Using Dual-Wavelength Surface Acoustic Wave Devices

**DOI:** 10.3390/mi10010052

**Published:** 2019-01-13

**Authors:** Jin-Chen Hsu, Chih-Hsun Hsu, Yeo-Wei Huang

**Affiliations:** Department of Mechanical Engineering, National Yunlin University of Science and Technology, Douliou, Yunlin 64002, Taiwan; q20a65x@yahoo.com.tw (C.-H.H.); gawain7373@gmail.com (Y.-W.H.)

**Keywords:** microfluidics, dual-wavelength SAW, acoustic radiation force, interdigital transducer

## Abstract

We present a numerical and experimental study of acoustophoretic manipulation in a microfluidic channel using dual-wavelength standing surface acoustic waves (SSAWs) to transport microparticles into different outlets. The SSAW fields were excited by interdigital transducers (IDTs) composed of two different pitches connected in parallel and series on a lithium niobate substrate such that it yielded spatially superimposed and separated dual-wavelength SSAWs, respectively. SSAWs of a singltablee target wavelength can be efficiently excited by giving an RF voltage of frequency determined by the ratio of the velocity of the SAW to the target IDT pitch (i.e., *f* = *c*_SAW_/*p*). However, the two-pitch IDTs with similar pitches excite, less efficiently, non-target SSAWs with the wavelength associated with the non-target pitch in addition to target SSAWs by giving the target single-frequency RF voltage. As a result, dual-wavelength SSAWs can be formed. Simulated results revealed variations of acoustic pressure fields induced by the dual-wavelength SSAWs and corresponding influences on the particle motion. The acoustic radiation force in the acoustic pressure field was calculated to pinpoint zero-force positions and simulate particle motion trajectories. Then, dual-wavelength SSAW acoustofluidic devices were fabricated in accordance with the simulation results to experimentally demonstrate switching of SSAW fields as a means of transporting particles. The effects of non-target SSAWs on pre-actuating particles were predicted and observed. The study provides the design considerations needed for the fabrication of acoustofluidic devices with IDT-excited multi-wavelength SSAWs for acoustophoresis of microparticles.

## 1. Introduction

In recent years, noninvasive and contactless manipulation of suspended micro-objects has garnered substantial interest [1,2,3,4]. Specifically, acoustic manipulation in microfluidic channels has emerged to serve as an effective tool in microfluidics to control micron-sized objects for chemical, physical and biological applications [5,6]. Pure and controllable acoustic forces involved in the acoustic manipulation method produce little or no damage to the viability and functionality of biological cells [7]. A trend in acoustic manipulation methods involves creating a standing acoustic-wave field across a microfluidic channel and employing the resulting acoustic pressure field to transport, trap, separate, pattern or sort microparticles or bio-cells suspended in microfluids [8,9,10,11,12,13,14,15]. Various types of acoustic-wave modes have been utilized to produce the desired acoustic pressure fields, including ultrasonic bulk acoustic waves (BAWs) [16,17,18,19,20,21], surface acoustic waves (SAWs) [22,23,24,25,26,27,28,29,30] and Lamb waves (LWs) [31,32,33]. With respect to the design of efficient and reproducible devices, increasing attention has been focused on SAW-based microfluidic devices owing to their simple geometry, precise definition of dimensions by micro-fabrication and easy integration with channels composed of soft polymers. 

With respect to SAW-based microfluidic devices, one of the major efforts has been focused on establishing a standing SAW (SSAW) field to radiate acoustic energy into microfluids to generate the desired driving forces on particles by forming a standing acoustic pressure field. Generally, suspended particles can be subject to two kinds of acoustic forces: acoustic radiation force (ARF) and the Stokes’ drag force [34,35]. ARF induced on particles in an acoustic field can be the result of both primary and secondary (Bjerknes) radiation forces, where the primary force originates from the acoustic field and the secondary forces are due to acoustic waves scattered by other particles [1,36]. On the other hand, the Stokes’ drag force is mainly due to the induced acoustic streaming flow (ASF). The particle motion driven by these acoustic forces is referred to as acoustophoresis. In the case of particles with sizes in the range from several µm to tens of µm and acoustic frequencies ranging from several MHz to tens of MHz, theoretical and experimental investigations have indicated that the ARF usually dominates over the ASF-induced Stokes’ drag force [36,37,38]. A clear demarcation between the regimes dominated by ASF or by ARF can be determined by the force balance criterion between ASF and ARF [34]. This criterion gives the critical particle diameter and when the particle diameter is larger than the critical value, the ARF dominates. Both ASF and ARF effects have been utilized to manipulate particles, cells or droplets in microfluids [39,40,41,42,43]. Moreover, SSAWs have been adopted in many acoustic manipulation schemes because the induced standing acoustic pressure can enhance the steadiness of the motion control using ARF for smaller-sized particles, such as separating mixed particles of different sizes or densities [44], sorting biological cells [45], patterning multiple spatially separated single particles and cells [46] and focusing particles at different locations in a channel by SAW phase shifting or frequency switching [47,48]. 

Multi-wavelength SAW devices have immense potential in a wide range of tunable applications for microfluidic devices [49,50,51]. Generally, conventional design simulation of SAW acoustofluidic devices assumed single-frequency SAW radiation on the fluidic domain boundary. A specific, well-controlled SAW frequency was assumed to be generated by applying a corresponding RF voltage at a specific frequency in real experiments. However, for SAWs excited using IDTs, considerable bandwidth of the SAW can be provided by the IDT even if the fingers are single pitched. Generally, the fewer fingers of a specific pitch in the IDT, the wider the SAW bandwidth. This could be an issue in the multi-pitch or chirped IDT when a specific frequency and phase of SAW needs to be excited, because there are only a few fingers for each pitch in the chirped IDT. RF voltage excitation of SAWs through multi-pitch/chirped IDTs on a piezoelectric substrate inevitably excites unwanted SAWs with close frequencies and/or deviated phases by other fingers of different pitches that may disturb the SSAW field intended to be defined by the target SAWs with a single frequency/wavelength and a specific phase. The effects of excited SAWs with non-target SAWs through the piezoelectric effect in a crystalline solid substrate on the resulting acoustic pressure fields in the case of multi-wavelength SSAW devices were not investigated by previous studies. To explore the effects of a tunable SSAW field on acoustophoresis from a more systematic design aspect, two dual-wavelength SSAW devices with two different IDT designs were employed in the present study. The two designs yield SSAWs that bring the effect of spatially superimposed and separated dual-wavelength SSAW fields and their influences on the particle motion in a microfluidic channel. Here, we present the numerical calculations using a coupled model of a fluid and a piezoelectric solid to demonstrate the design process and to comprehensively study the effects involved. Experimental results are then presented and discussed to demonstrate the effective control of particle transport that results from implementing these design considerations. 

## 2. Methods

### 2.1. Acoustofluidic Theory and Numerical Models 

**Figure 1:**
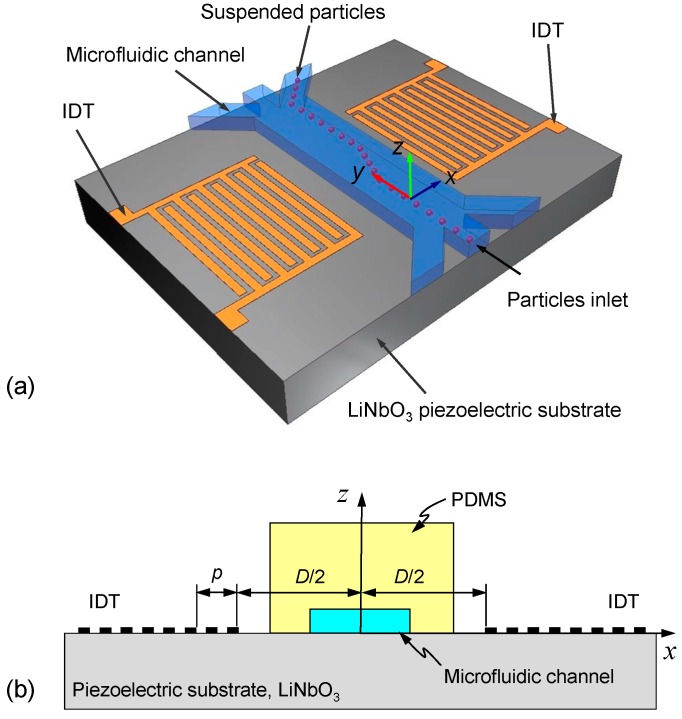
(**a**) Configuration of a standing surface acoustic wave (SSAW)-based acoustofluidic device with single-pitch interdigital transducers (IDTs) and (**b**) the cross-section model for the numerical simulations (illustrating the symmetric structure). The device consists of a microfluidic channel with three inlets and three outlets bonded to a piezoelectric substrate patterned with two IDTs. Suspended particles in the channel can be manipulated to exit through a specific outlet.

Figure 1a shows the configuration of a SSAW-based acoustofluidic device. The device consists of a microfluidic channel bonded to a piezoelectric substrate patterned with two interdigital transducers (IDTs). The microfluidic channel is composed of polydimethylsiloxane (PDMS) and is positioned between the two IDTs. When an RF voltage is applied to the IDTs to generate two series of identical SAWs that propagate in counter directions toward the microfluidic channel, they interfere to generate a SSAW field in the area in which the microfluidic channel is bonded. While the SSAWs transmit along the fluid/solid interface, radiation of longitudinal acoustic waves into the fluid encapsulated in the channel occurs and causes pressure fluctuations inside the fluid. As a result, particles suspended in the fluid are subjected to acoustic forces due to the acoustic radiation and pressure fluctuations, which allow manipulation of the microparticles. 

In order to study the acoustophoretic effects of suspended particles in the channel using the device, a two-dimensional cross section model of the device is considered as shown in Figure 1b. The constitutive law of a piezoelectric material can be expressed as follows [52,53]:

(1) $$T=c^{E}:S-e\cdot E,\;$$

(2) $$D=e:S+\varepsilon^{S}\cdot E,\;$$

where **T** and **S** denote stress and strain tensor fields, respectively; **c***^E^* denotes an elastic stiffness tensor of rank four at constant electric field; **E** and **D** denote the electric vector field and electric displacement vector field, respectively; **ε***^S^* denotes a permittivity tensor of rank two at constant strain; and **e** denotes a piezoelectric tensor of rank three. The electric field **E** is assumed to be quasi-static and related to the electric potential field *φ* according to: **E** = –gradient *φ*. The governing field equations of time-harmonic acoustic vibration in the piezoelectric material are given by the following expressions:

(3) $$\nabla \cdot T=-\omega^{2}\rho_{\mathrm{LN}}u-b,$$

(4) ∇⋅D=0, 

where **u** denotes the mechanical displacement vector field, **b** denotes the body force vector (force/volume) in the material, *ρ*_LN_ denotes the mass density of the piezoelectric substrate (lithium niobate, LiNbO_3_, is used here) and *ω* denotes the acoustic angular frequency. The relation between the mechanical displacement and strain can be expressed as follows:

(5) $$S=\frac{1}{2}\left(\nabla u+{\left(\nabla u\right)}^{T}\right).\;$$

In the microfluid and PDMS domains, the acoustic pressure field *P* for a linearly elastic fluid with attenuation is governed by the Helmholtz wave equation as given below [54]:

(6a) $$\nabla \cdot \left(-\frac{1}{{\bar{\rho }}_{\left(i\right)}}\nabla P\right)-\frac{\omega^{2}}{{\bar{\rho }}_{\left(i\right)}{\bar{c}}_{\left(i\right)}^{2}}P=0,\;$$

where

(6b) $${\bar{\rho }}_{\left(i\right)}=\frac{\rho_{\left(i\right)}c_{\left(i\right)}^{2}}{{\bar{c}}_{\left(i\right)}^{2}},\;{\bar{c}}_{\left(i\right)}=\frac{\omega }{k_{\left(i\right)}},\;k_{\left(i\right)}=\frac{\omega }{c_{\left(i\right)}}-j\alpha_{\left(i\right)}.\;$$

In Equation (6b), *ρ*_(*i*)_, *c*_(*i*)_ and *α*_(*i*)_ denote the mass density, speed of sound and attenuation coefficient in the corresponding domain (*i*), respectively; the subscript (*i*) denotes the microfluid or PDMS and *j* denotes the imaginary number $\sqrt{-1}$. The velocity field (denoted as **v**) of the fluid caused by the small acoustic variation is given by the following expression:

(7) $$v=-\frac{\nabla P}{j\omega {\bar{\rho }}_{\left(i\right)}}.\;$$

When using the Helmholtz wave equation, acoustic streaming in the fluid is assumed to be omitted because ARF dominates the particle motion in this SSAW scheme. 

Electric actuation through the piezoelectric effect is used to generate SAWs. Actuation was performed by imposing a sinusoidal electric potential of some particular frequency (denoted as *ω*) on the IDT electrodes by connecting the IDTs to an external voltage source of the form *φ* = *V*_0_*e^jωt^*, where *V*_0_ denotes voltage amplitude. According to Equations (1) and (2), the applied voltage produces sinusoidal stress and strain fields in the piezoelectric substrate near the IDT areas and develops SAWs of wavelengths associated with the IDT pitches. 

The coupling between acoustic pressure fields in the microfluid and PDMS and the SSAW field in the piezoelectric substrate is controlled by the mechanical boundary conditions at the fluid/solid interfaces. The shear wave propagation is ignored in PDMS given its significant attenuation and negligible coupling due to weak conversion from longitudinal waves or acoustic pressure [55]. Additionally, it is assumed that there is no energy radiation out of the device domain. This involves the assumption of a surrounding vacuum by applying the condition of a prefect reflection from the PDMS surface. The mechanical vibration of SAWs applies a force load on the interface where the piezoelectric substrate is in contact with the microfluid and PDMS. The interface boundary condition provides continuity between stress and acoustic pressure in the normal direction as follows:

(8) (n⋅T⋅n) n=−Pn, 

where **n** denotes the unit vector normal to the interface boundaries. The force load given by Equation (8) serves as the source of longitudinal leaky waves into the microfluid and PDMS to build an acoustic pressure field in the microfluidic channel. The continuity of the normal component of the acceleration (denoted by *a_n_*) is applied along the interface of the microfluid and PDMS as follows:

(9) $$\left(-\frac{\nabla P}{{\bar{\rho }}_{f}}\right)\cdot n=\left(-\frac{\nabla P}{{\bar{\rho }}_{\mathrm{PDMS}}}\right)\cdot n=a_{n}.\;$$

The acoustophoretic motion of *N* suspended particles in the microfluidic channel is determined by the forces acting on the particles. According to Newton’s second law, the motion of *q*-th suspended particle is governed by the following expression:

(10) $$m_{q}\frac{dv_{q}}{dt}=\sum_{k}F_{k}(r_{q}),\;(\mathrm{for}\;q=1\;\mathrm{to}\;N),\;$$

where *m_i_*, **v***_q_* and **r***_q_* denote the mass, velocity and position of the *q*-th particle; **F***_k_*(**r***_q_*) denotes the force acting on the particle at position **r***_q_*; and the subscript *k* denotes different force sources. The rotational motion of the particles is ignored by assuming that the particles are spherical and sufficiently small [56]. In a microfluid with an acoustic pressure field, the time-averaged acoustic radiation force (ARF), **F**_rad_, exerted on a small particle of radius *a* is given by the following expression [56,57]:

(11) $$F_{\mathrm{rad}}=-\pi a^{3}\left[\frac{2}{3\rho_{f}c_{f}^{2}}\left(1-\frac{1}{\tilde{\rho }{\tilde{c}}^{2}}\right)\nabla \langle P^{2}\rangle -\frac{2\tilde{\rho }-2}{2\tilde{\rho }+1}\rho_{f}\nabla \langle {\left|v\right|}^{2}\rangle \right],\;$$

where $\tilde{\rho }=\rho_{p}/\rho_{f}$ and $\tilde{c}=c_{p}/c_{f}$ denote the mass-density ratio and speed-of-sound ratio, respectively. The subscript p denotes the particle. In Equation (11), we adopt the real-valued result given by Gor’kov when considering an immersed particle of radius *a* that is much smaller than the acoustic wavelength in the fluid; and we neglect the fluid viscosity. More general expressions of ARF that take into account the general scattering coefficients based on standard scattering theory of a small particle in viscous fluid can be found in Refs. [58,59]. The drag force (denoted by **F**_drag_) on the particle due to its motion in the *xz*-plane relative to the fluid with ASF being neglected is given by the following [34,60]:

(12) $$F_{\mathrm{drag}}=-6\pi \eta av_{q},$$

where *η* denotes the dynamic viscosity of the fluid. In addition, the gravitational force (denoted by **F***_g_*) and buoyant force (denoted by **F***_B_*) also act on the particles oppositely in the *z*-direction. 

### 2.2. Device Design Approach

**Figure 2:**
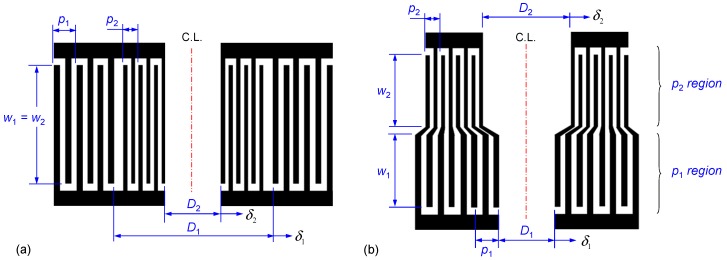
(**a**) Parallel and (**b**) series configurations of IDT designs for generating switchable dual-wavelength SSAW fields. In the parallel (series) IDT design, IDTs of two different pitches (denoted as *p*_1_ and *p*_2_) are connected in parallel (series).

In the present study, two different configurations of IDT designs are proposed, namely *parallel* and *series* designs to generate switchable dual-wavelength SSAWs. Figure 2 shows the geometries of the two designs. With respect to the parallel (series) IDT design, IDTs of two different pitches (denoted as *p*_1_ and *p*_2_) are connected in parallel (series). The design parameters are listed in Table 1. When a single-frequency RF voltage is applied to the two-pitch IDTs, SAWs of a specific wavelength (e.g., *λ*_1_ ≅ *p*_1_) can be effectively excited if the input frequency *f* satisfies the condition: *f* = *f*_1_ ≅ *c*_SAW_/*p*_1_, where *c*_SAW_ denotes the velocity of the SAW. These SAWs are referred to as the “target SAW”, and they typically have higher intensity. However, the target SAWs may be accompanied by non-target SAWs with lower intensity and at other wavelengths (typically: *λ*_2_ ≅ *p*_2_ ); in general, the non-target SAWs are not effectively excited. Although both IDT designs consist of the same two pitches (that is, *p*_1_ and *p*_2_), the dual-wavelength SAWs (target SAWs and non-target SAWs) excited by the parallel IDTs are spatially superimposed along the propagating path through the microfluidic channel while those excited by the series IDTs are spatially separated. The acoustic pressure fields are further designed to produce a switchable pressure node in the channel to demonstrate the microparticle transport function. Particles flowing along the channel are laterally displaced toward the pressure node by net forces from the acoustic and flow fields and the location of the pressure node can be switched by changing the input frequency to transport the suspended particles toward the desired outlet. The following section explores and discusses the tunable acoustophoretic effects and the influences of different SSAW fields excited by the proposed IDT designs through numerical and experimental investigations.

### 2.3. Device Fabrication and Experimental Setup

The device was fabricated on a 128° *Y*/*X* LiNbO_3_ wafer with a thickness of 500 μm. The propagation direction of the excited SAWs was set such that it aligned with the crystalline *X* axis. This wafer was selected as the substrate because of its high electromechanical coupling coefficient (*K*^2^ ≅ 5.3%) for SAWs. The IDTs were fabricated using a microfabrication technique. First, an aluminum (Al) film of 0.1 μm thickness was deposited on the wafer using an e-beam evaporator. This was followed by spin coating a 2 μm thick positive photoresist (AZ-1500, Merck, Darmstadt, Germany) on the Al film and prebaking at 95 °C for 2 min. Photolithography and development were employed to transfer the IDT patterns from a predesigned mask onto the photoresist. The IDT electrodes were defined by wet etching the unwanted area of the Al film and removing the developed photoresist. The PDMS microfluidic channel that contains three inlets and three outlets at each of its two ends was fabricated through a soft-lithography and mold-replica procedure. The mold for casting the PDMS channel was fabricated by inductively-coupled plasma (ICP) deep etching of a pre-patterned and masked silicon wafer. Finally, the PDMS microfluidic channel and the LiNbO_3_ wafer were surface-activated by oxygen plasma and were then carefully aligned and bonded.

For the experiments, polystyrene microparticles (diameter 2*a* = 8 μm and specific gravity *γ* = 1.05) suspended in water were injected into the channel through the central inlet and pure water was injected simultaneously through the two outer inlets as the sheath flowed to centralize the particles and prevent direct adhesion of the particles on the sidewall of the channel. These flows were injected using a syringe pump with controlled flow rates. The images of acoustophoretic particle motion were captured using a camera with a maximum frame rate of 200 frames/s mounted on an optical microscope. An RF signal generated by a signal generator (Agilent 5181A, Keysight, Santa Rosa, CA, USA) was amplified by a power amplifier (Amplifier Research 75A250A, Amplifier Research, Souderton, PA, USA). The amplified RF signals were applied to the two IDTs and converted into two identical SAW beams transmitting toward the channel. The frequency responses of the fabricated dual-wavelength SAW devices were determined using a network analyzer (Agilent E5061B, Keysight, Santa Rosa, CA, USA).

## 3. Results and Discussion 

### 3.1. Design Details, Simulation Results and Discussion

The numerical calculations for the SSAW acoustofluidic model were performed using a finite element solver package COMSOL Multiphysics [61]. Table 2 lists the material parameters used in the numerical calculations. To clearly demonstrate the dual-wavelength effect in the device, the simulation commenced with considering a structure symmetric to the *z*-axis as illustrated in Figure 1b. The PDMS with a microfluidic channel filled with water was placed in the middle of a pair of two-pitch parallel IDTs [*p*_1_ = 398 μm and *p*_2_ = 363 μm in Figure 2a]. The dimensions included *w* × *h* = 1600 μm × 1000 μm for the PDMS and 250 μm × 25 μm for the embedded microfluidic channel, where *w* and *h* denote width and height, respectively. Additionally, *D*/2 denotes the distance of the electrode of the left- or right-hand IDT from the *z*-axis and *D* denotes the delay length to provide a sufficient space between the electrodes. Based on the criterion of force balance between ASF and ARF, the critical particle diameter under our operating conditions is estimated to be about 0.8–1.4 μm. For modeling purposes, a non-reflecting boundary condition was applied at the bottom, left and right sides of the LiNbO_3_ substrate to prevent the reflection of out-going waves. At the top surface of the substrate where the SAWs were guided, a stress-free boundary condition was specified. In the region where the top surface of the substrate is in contact with the PDMS and fluid, continuity conditions were satisfied according to Equations (8) and (9). To achieve convergent results, the maximum mesh size *d*_mesh_ is limited by the condition *d*_mesh_ < *λ*_i_/20, where *λ*_i_ is the acoustic wavelength of the relevant frequency in the corresponding domain. As a result, the numbers of mesh elements in the LiNbO_3_, water and PDMS domains were 1,341,531, 1,146,480 and 1,323,693, respectively, in the calculations. 

**Figure 3:**
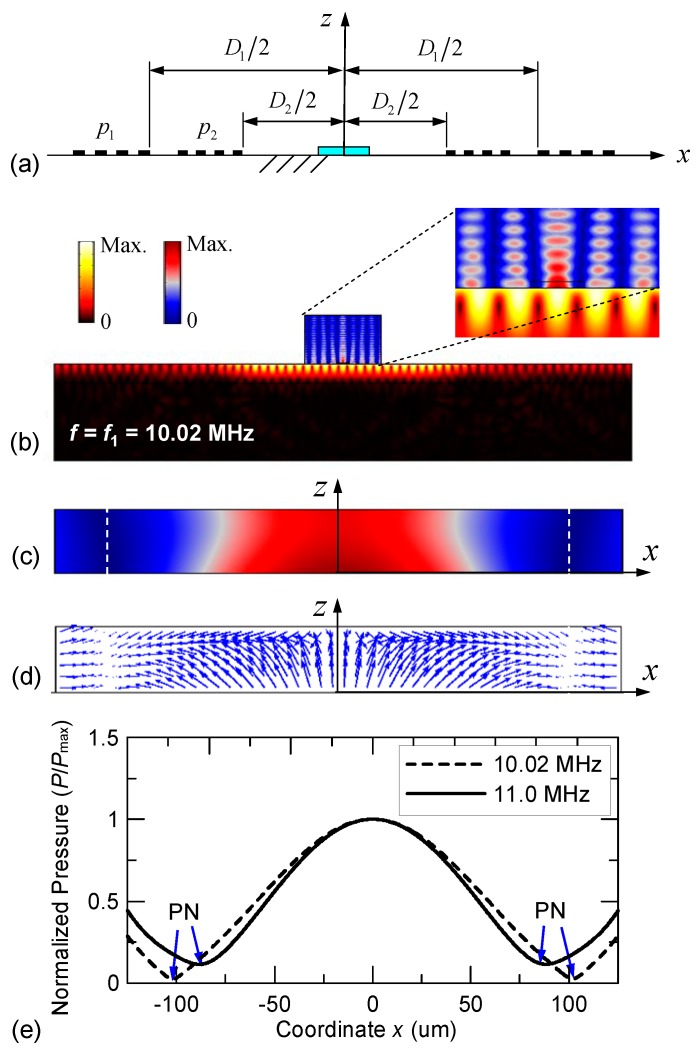
(**a**) Schematic of the symmetric two-pitch IDT arrangement on the surface of the LiNbO_3_ substrate. The IDT pitches are *p*_1_ = 398 μm and *p*_2_ = 363 μm. Simulated results of (**b**) SSAW total displacement field and acoustic pressure field in the entire device generated by a driving voltage at 10.02 MHz and (**c**) normalized acoustic pressure field and (**d**) acoustic radiation force field in the water domain. (**e**) Comparison of the normalized acoustic pressure variations using 10.02 MHz and 11.0 MHz SSAW fields along the width direction of the water domain at *z* = 12.5 μm. (The acronym PN stands for “pressure node.”).

Figure 3a,b show the schematic of the symmetric two-pitch arrangement on the top surface of the LiNbO_3_ substrate and the simulated results. The frequency of the applied voltage is *f*_1_ = 10.02 MHz. At steady state, the two series of dual-wavelength SAWs simultaneously excited by the left- and right-hand IDTs interfere to produce SSAWs in the central region of the LiNbO_3_ substrate and radiate longitudinal waves into the PDMS and water domains to create a standing acoustic pressure field. Figure 3c,d show the calculated acoustic pressure and ARF fields in the water domain. The acoustic pressure field exhibits a symmetric profile with two acoustic pressure nodes (PNs) that correspond to two zero-force points in the symmetric ARF field. The force field also indicates actuating directions toward the pressure nodes. This reveals that the acoustic pressure nodes locate around the positions of the stationary points in the channel for the motion of suspended particles. Figure 3e shows the comparison of the variations of the acoustic pressure along the *x* direction and at the half height (i.e., at *z* = 12.5 μm) of the water domain when the frequency of the applied voltage is changed to *f*_2_ = 11.0 MHz. The symmetric acoustic pressure nodes are shifted towards the center (the *z* axis) by approximately 17 μm when the input frequency is changed from *f*_1_ to *f*_2_.

**Figure 4:**
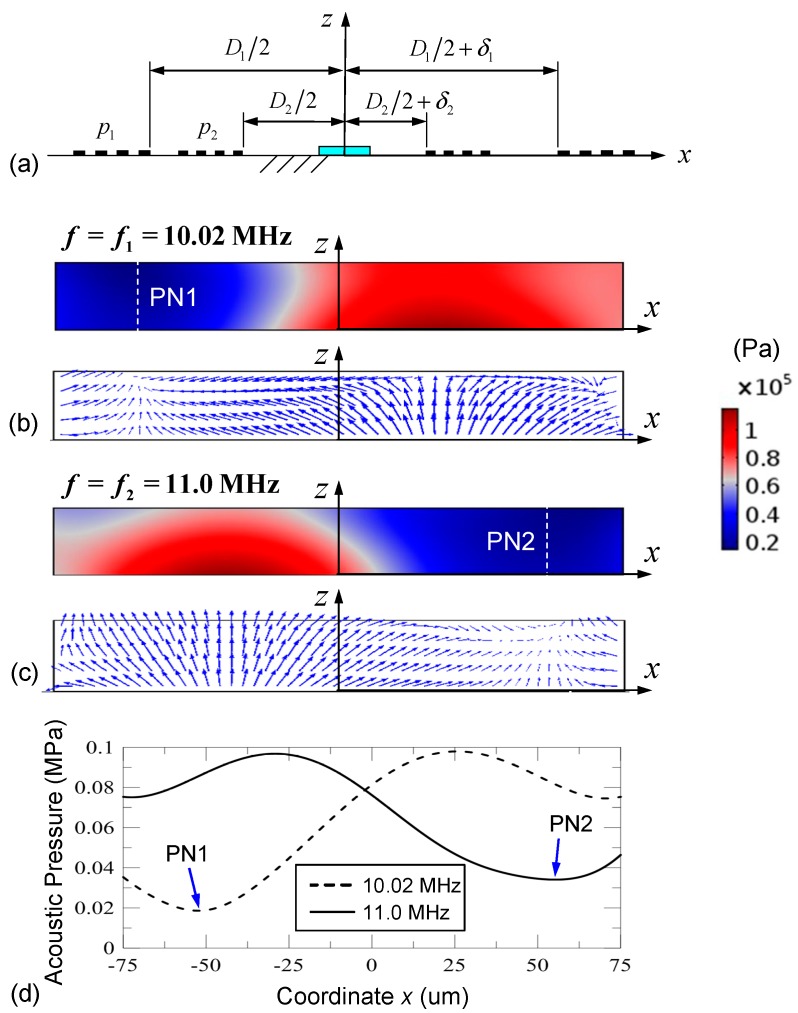
(**a**) Schematic of the two-pitch parallel IDT on the top surface of the LiNbO_3_ substrate. The IDT pitches are *p*_1_ = 398 μm and *p*_2_ = 363 μm. The translation distances of the right-hand parallel IDT are *δ*_1_ = 25 μm (rightward) and *δ*_2_ = –29 μm (leftward) to break the symmetric arrangement and form asymmetric field distributions. Simulated acoustic pressure and ARF fields generated by the SSAW field with the two-pitch parallel IDTs using frequencies of (**b**) *f*_1_ = 10.02 MHz and (**c**) *f*_2_ = 11.0 MHz at a voltage of *V*_0_ = 16 V. (**d**) PN locations and comparison of the acoustic pressure variations using *f*_1_ = 10.02 MHz and *f*_2_ = 11.0 MHz along the width direction of the water domain.

The two-pitch IDTs for dual-wavelength SSAWs were then rearranged to design controllable motion of the particles to exit through a desired outlet by switching the position of the pressure nodes. This can be achieved by breaking the symmetry of the IDT locations and reducing the channel width to 150 μm. The parallel IDTs were first considered in which IDTs with *p*_1_ and *p*_2_ were connected in parallel. The translation distances in the right-hand parallel IDT are *δ*_1_ = 25 μm and *δ*_2_ = −29 μm (minus sign means a leftward translation), as shown in Figure 4a. Figure 4b–d show the simulated results with a voltage of *V*_0_ = 16 V and a frequency *f*_1_ = 10.02 MHz (or *f*_2_ = 11.0 MHz) applied to the parallel IDTs. Asymmetric distributions of the acoustic pressure and ARF fields are formed. A single pressure node (PN1) appears at the left side of the water domain (52 μm from the *z*-axis) when the frequency of the applied voltage is *f*_1_ = 10.02 MHz. As shown at PN1 in Figure 4b, the ARF field has zero horizontal components and nonzero vertical components. The nonzero vertical components are a result of the nonzero gradient of the acoustic pressure field along the *z*-direction and are intensified in the spatially superimposed dual-wavelength SSAW field. When the input frequency was changed to *f*_2_ = 11.0 MHz, a pressure node (PN2) appears at the right side of the water domain (55 μm from the *z*-axis) as shown in Figure 4c. Figure 4d compares the acoustic pressure variations in the water along the *x* direction when two different frequencies are applied to the two-pitch parallel IDTs. The results show that the difference in the two SAW wavelengths results in a non-fully mirror symmetry of the acoustic pressure field with respect to the *z*-axis and the left and right sidewalls significantly modulate the pressure field. The points referred to here as “pressure nodes” are the local minima of the acoustic pressure, rather than places where the pressure definitely goes all the way to zero as would be expected in an ideal SSAW situation. The nonzero pressure value of the pressure node originates from two effects: (1) oblique radiation of SSAWs into the fluid and (2) interference of dual-wavelength SSAWs. The acoustic pressure fields were significantly modulated in the parallel IDT design as a result of the second effect. Shifting the IDTs enhances the second effect and reduces the pressure variations across the channel width. Superimposed dual-wavelength SSAWs perturb not only the pressure magnitude but also the pressure node locations from those of SSAWs produced by single-wavelength SAWs and could increase the uncertainty for predicting the location of the SSAW pressure nodes. 

**Figure 5:**
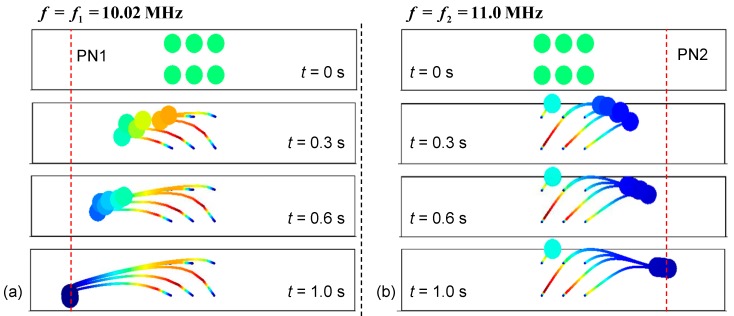
Calculated particle trajectories in the parallel IDT design using two different input frequencies: (**a**) *f*_1_ = 10.02 MHz and (**b**) *f*_2_ = 11.0 MHz. An array of six particles (8 μm in diameter) are released from the central region of the channel at time *t* = 0 s.

Figure 5 shows the particle trajectories calculated on the basis of the acoustic pressure fields with the two different input frequencies. An array of six particles (8 μm in diameter, which is much larger than the estimated critical particle diameter) is released from the central region of the channel at a time corresponding to *t* = 0 s. When the input frequency (*f*_1_) is 10.02 MHz, the particles are driven toward PN1 and reach PN1 prior to *t* = 1.0 s. Initially, levitation of the particles is observed because of the considerable vertical component of the ARF and then the particles descend because of gravitational force. Note that the gravitational force is not completely cancelled by the buoyant force. The net effect of **F**_g_ and **F**_B_ is about 0.1–1 pN. In contrast, when the input frequency is *f*_2_ (11.0 MHz), the particles are driven toward PN2 and reach PN2 within *t* = 1.0 s in a similar manner. However, the vertical levitation is more obvious when moving to PN2 and particles halt at PN2 with a higher altitude as a result of spatial superposition of the dual-wavelength SAWs. In the figures showing the particle trajectories, the colors in the particle trajectories represent the particle speed. Red and blue colors represent higher and lower speed, respectively.

**Figure 6:**
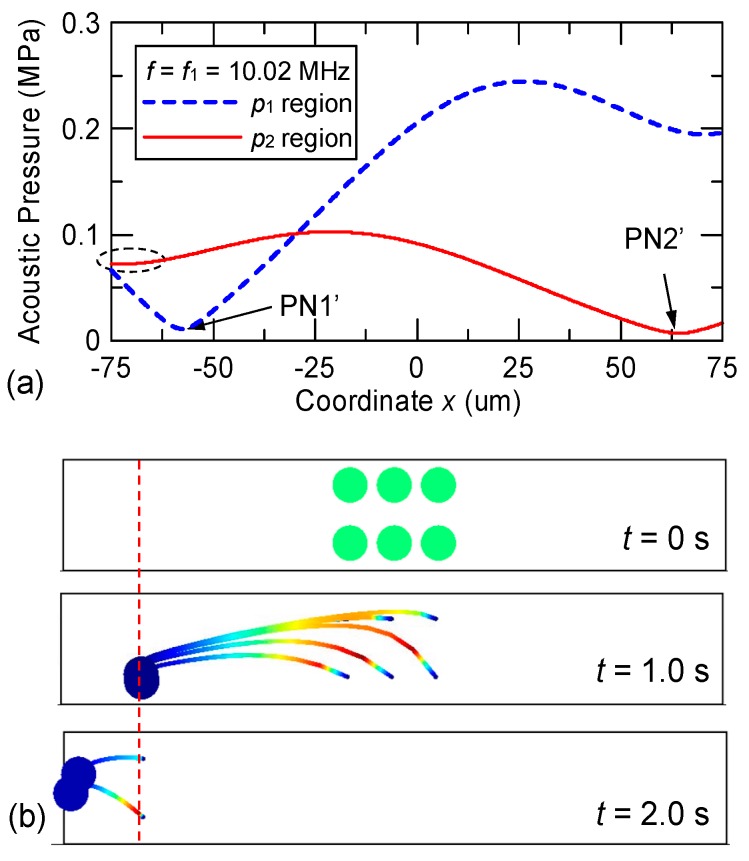
(**a**) Calculated acoustic pressure variations and (**b**) particle trajectories in the design of the series IDTs using the input frequency *f*_1_ = 10.02 MHz in the *p*_1_ and *p*_2_ regions.

Considering the series IDTs [see Figure 2b], the excited dual-wavelength SAWs are spatially separated along the microchannel length; and the acoustic pressure field is decomposed into two regions (denoted as *p*_1_ and *p*_2_ regions) corresponding to the regions of SAW propagation excited by IDTs with pitches *p*_1_ = 398 μm and *p*_2_ = 363 μm, respectively. Figure 6a shows the simulated results when the frequency of the input voltage to the series IDTs is *f*_1_ = 10.02 MHz. Within the *p*_1_ region, the pressure variation is indicated by the dashed line in Figure 6a and a pressure node (PN1′) appears at the left side of the water domain. Particles released in the *p*_1_ region from the central part of the water domain are driven to PN1′ within 1.0 s, as shown in the middle panel of Figure 6b. The particle tracing calculation is continued by assuming that the particles enter the *p*_2_ region at *t* = 1.0 s. In the *p*_2_ region, the solid line in Figure 6a indicates the pressure variation and the pressure intensity is considerably smaller than that in the *p*_1_ region because of the ineffective excitation of SAWs through the IDTs of pitch *p*_2_ using a voltage at 10.02 MHz. However, in the series IDT design, the possibility of actuation by the non-target SSAWs may arise because a pressure node (PN2′) appears at the right side of the water domain and a local low-pressure area is formed near the left sidewall in the *p*_2_ region. The left local low-pressure area further drives the particles (post-actuation) toward the sidewall as well as preventing a reversal of the particles, as shown by the particle trajectories in the bottom panel of Figure 6b. 

**Figure 7:**
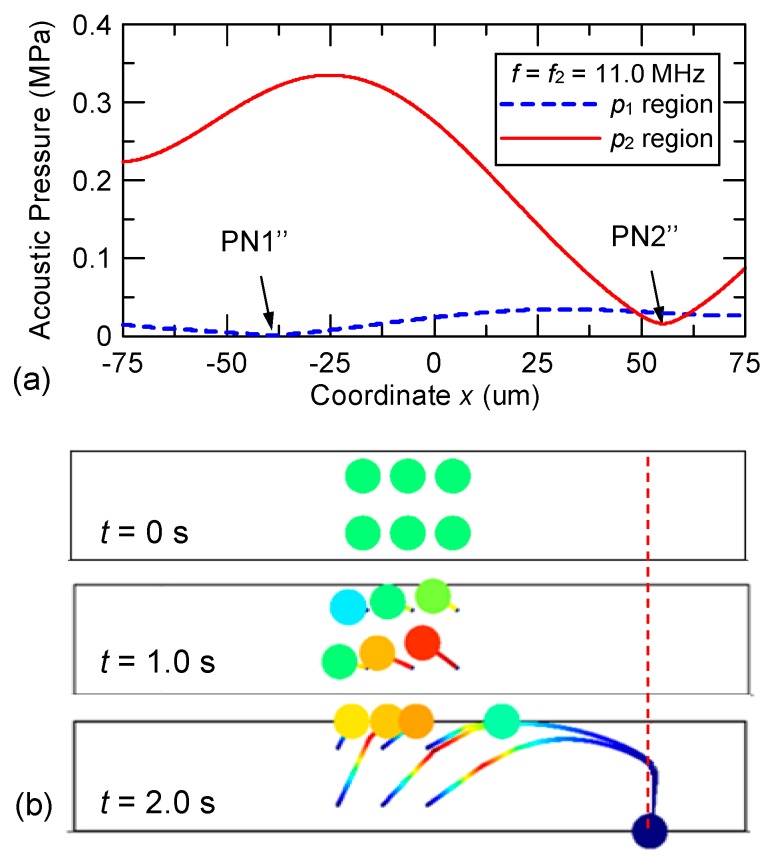
(**a**) Calculated acoustic pressure variations and (**b**) particle trajectories in the design of the series IDTs using the input frequency *f*_2_ = 11.0 MHz in the *p*_1_ and *p*_2_ regions.

Figure 7 shows the simulated results when the frequency of the input voltage applied to the series IDTs is *f*_2_ = 11.0 MHz. Within the *p*_1_ region, the variation of acoustic pressure is mild and the intensity is low because of the ineffective excitation of 398 μm SAWs; the variation is indicated by the dashed line in Figure 7a where a pressure node (PN1′′) appears at the left side of the water domain. However, this pressure field produced by the non-target SSAWs in the *p*_1_ region was still able to drive the particles toward left; here we refer to this effect as “pre-actuation.” In order for the design to perform properly, the left displacement by the pre-actuation in the *p*_1_ region caused by the non-target SSAWs must be small enough so that the acoustic pressure of the *p*_2_ region will still be able to reverse the particles toward PN2.” As shown in the middle panel of Figure 7b, particles released in the *p*_1_ region are slowly driven toward PN1′′ during a time period corresponding to 1.0 s (pre-actuation). Then the particle tracing calculation is continued by assuming that the particles enter the *p*_2_ region at *t* = 1.0 s as a result of the continuous flow. In the *p*_2_ region, the 363 μm SAWs are effectively excited and result in a drastic pressure variation as shown by the solid line in Figure 7a. A pressure node (PN2′′) appears at the right side of the water domain and reverses the drift direction of the slightly-leftward-shifted particles so that they start to move toward PN2′′ as shown by the particle trajectories in the bottom panel of Figure 7b.

### 3.2. Experimental Results and Discussion

**Figure 8:**
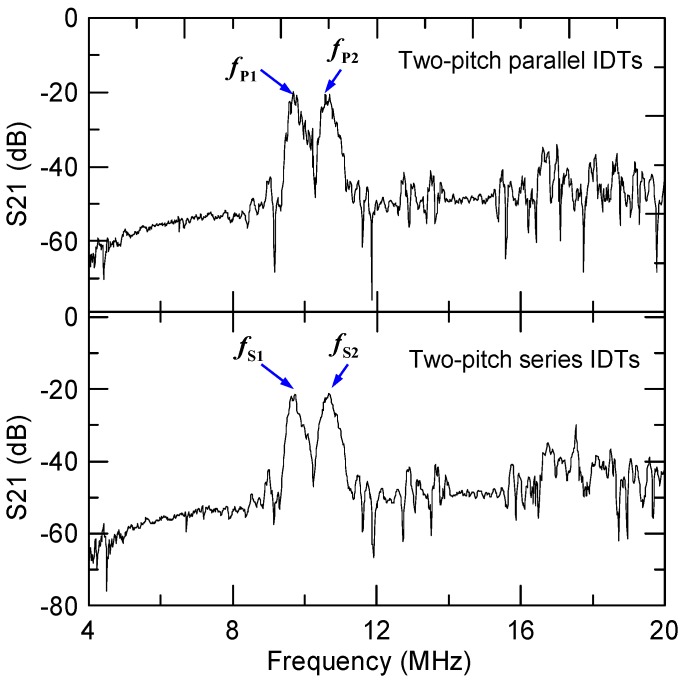
Measured frequency-domain responses (Scattering parameter S_21_ using a network analyzer) of the two-pitch parallel and series IDTs fabricated on the LiNbO_3_ wafer. Two frequency peaks are observed in each design of parallel or series IDTs. *f_P_*_1_ = 9.68 and *f_P_*_2_ = 10.56 MHz; *f_S_*_1_ = 9.73 and *f_S_*_2_ = 10.65 MHz.

In Section 3.1, we discussed the theory of the transport control of a particle by switching the acoustic frequency using the parallel and series IDTs. The dual-wavelength SSAWs excited by the IDTs can produce distinct acoustic pressure fields in the water domain confined by the channel. In order to achieve transport control, the bandwidth of the excited SAWs has to be narrow enough to reduce the non-target SSAWs and approximate a well-defined SSAW field. Therefore, excitation quality of SAWs by the device with a single-frequency RF voltage is required. We used 20 pairs in the IDT for each wavelength and the measured frequency responses (Scattering parameter S_21_) of the two-pitch parallel and series IDTs fabricated on a 128° *Y*/*X* LiNbO_3_ wafer are shown in Figure 8. Note that in addition to S_12_ signal that determines the transmission frequency response of IDTs, S_11_ signal is also important for understanding the reflection responses. Generally, the energy efficiency of SAW devices can be improved by considering more advanced designing methods [62]. In Figure 8, two peak frequencies are observed in each curve. For the parallel IDTs, the two peak frequencies are *f_P_*_1_ = 9.68 and *f_P_*_2_ = 10.56 MHz. For the series IDTs, the two peak frequencies are *f_S_*_1_ = 9.73 and *f_S_*_2_ = 10.65 MHz. The small deviation between the measured and theoretical frequencies could arise from the difference of material properties between the real LiNbO_3_ wafer and the calculation inputs. The measured peak frequencies were used in the acoustophoresis experiments as the input frequencies. The lower measured frequencies of SSAWs produce a longer distance between the left-side and right-side pressure nodes in the water domain in the experimental results given that lower frequencies correspond to longer acoustic pressure wavelengths. Another important observation on these frequency responses is that the two frequency peaks are clearly separated in both IDT designs, which implies the excitation of target SAWs is more effective with the target frequency than that of non-target SAWs. The excitation property is especially relevant to the change of acoustic pressure in the parallel IDT design, because the target and non-target SSAWs are superimposed in space and influence particle motion in the SSAW actuation region. 

**Figure 9:**
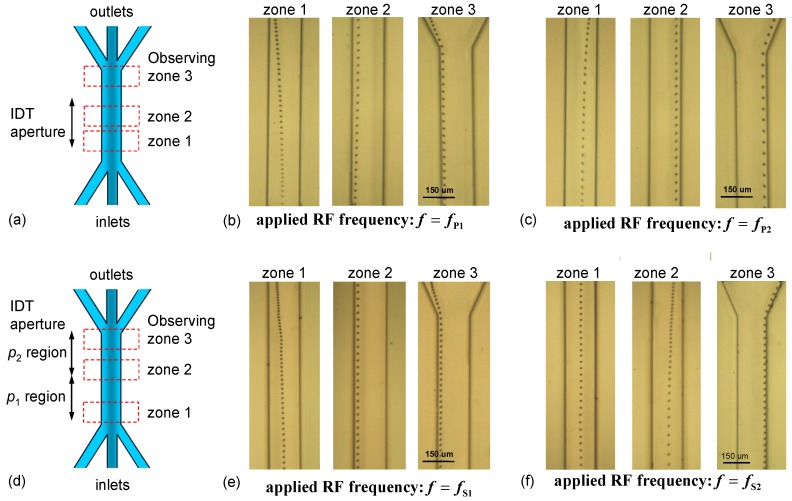
Schematic of the SSAWs working range covered by the (**a**) parallel and (**d**) series IDT apertures and three observing zones along the microfluidic channel. Stacked trajectories of a particle driven by the dual-wavelength SSAWs excited by the parallel IDTs at input frequencies (**b**) *f_P_*_1_ and (**c**) *f_P_*_2_ and by the series IDTs at input frequencies (**e**) *f_S_*_1_ and (**f**) *f_S_*_2_.

Figure 9a–c show the stacked trajectories of a particle driven by dual-wavelength SSAWs excited by parallel IDTs at *f_P_*_1_ and *f_P_*_2_, respectively. The input RF power was 30 dBm. The injecting volume flow rates of the sheath flow (water) and particle-suspended flow (particles in water) were 0.4 and 0.2 μL/min, respectively. The images were recorded at three areas (zones 1, 2 and 3) around the starting and middle points of SSAW working area and around the outlet, to clearly observe the particle motion. The results indicate that the particles were transported into the left (right) outlet by switching the input RF frequency to *f_P_*_1_ (*f_P_*_2_), respectively. The particles were laterally displaced to the corresponding pressure node (PN1 or PN2) within a short traveling distance along the channel and were then steadily guided by the forward laminar flow streamline and pressure node into the corresponding outlet. For the parallel IDT design with superimposed dual-wavelength SSAWs, the lateral displacements started in zone 1 and were completed before the particle entered zone 2. 

Even though the frequency difference Δ*f_P_* between *f_P_*_1_ and *f_P_*_2_ is as small as 0.88 MHz, Figure 8 shows that the two SAW resonance peaks are unambiguously distinguishable; and in Figure 6 and Figure 7, the corresponding non-target SSAWs in the design do not adversely affect the acoustic pressure nodes produced by the two SAW frequencies. Figure 9d–f, on the other hand, show the results of using the series IDTs, in which the SSAWs with frequencies *f_S_*_1_ and *f_S_*_2_ effectively work in the *p*_1_ and *p*_2_ regions, respectively, while the corresponding non-target SSAWs could be influential in the *p*_2_ and *p*_1_ regions, respectively. For *f* = *f_S_*_1_ [Figure 9e], the particles were driven to bend left toward PN1′ in zone 1 and move toward the left outlet. In contrast, for *f* = *f_S_*_2_ [Figure 9f], the particles were driven to PN2′′ and exited through the right outlet. When *f* = *f_S_*_2_, the particles evidently drifted rightwards until they entered the *p*_2_ region [see the image at zone 2 of Figure 9f] because of the ineffective SAW excitation in the *p*_1_ region. Even though the target and non-target SSAWs in the series IDT design are spatially separated, the ineffective excitation shown in Figure 6 and Figure 7 led to a much lower non-target SSAW intensity that, in turn, reduced the pre- and post-actuation influences. 

**Figure 10:**
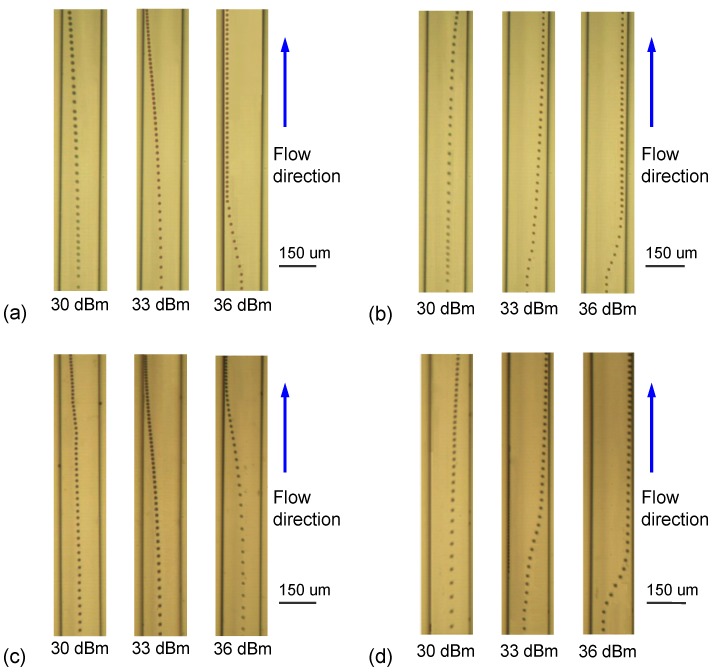
Experimental trajectories of the lateral displacement of a particle along the microfluidic channel with input RF power levels corresponding to 30 dBm, 33 dBm and 36 dBm in the design of parallel IDTs with input frequency (**a**) *f_P_*_1_ and (**b**) *f_P_*_2_; and in the design of series IDTs with input frequency (**c**) *f_S_*_1_ and (**d**) *f_S_*_2_.

Although increasing the input power does not change the ARF distribution in the model on the *xz*-plane, it increases the ARF magnitude in Equation (11) and leads to more rapid lateral motion along the *x* direction of the particle. To validate this effect experimentally, we varied the input RF power and observed the variation of the lateral displacement for the two-pitch IDT designs; the measured results on the *xy*-plane are shown in Figure 10. The flow rates of the sheath flow and particle-suspended flow were maintained at 0.4 and 0.2 μL/min, respectively. The time for the particles to travel through the recording zone was about 1.0 s. In the parallel IDT design with *f* = *f_P_*_1_ or *f* = *f_P_*_2_ [Figure 10a,b], the lateral displacement responds as expected and accelerates as a result of increasing the input RF power. The leftward and rightward trajectories are not fully symmetric because of the asymmetric acoustic pressure field produced by the two target frequencies. In the series IDT design [Figure 10c,d] there is once again an asymmetric trajectory depending on whether *f_P_*_1_ or *f_P_*_2_ is used, as well as the expected acceleration of lateral displacement with increasing RF power. A more interesting observation, however, is that the pre-actuation becomes more obvious when the input RF power is increased up to 33 and 36 dBm with *f* = *f_S_*_2_. Obvious leftward displacements occur before the particles enter the *p*_2_ region, as shown in Figure 10d. The leftward drift from the central line of the channel caused by pre-actuation of non-target SSAWs in the *p*_1_ region did not cross the anti-node of acoustic pressure in the *p*_2_ region; consequently, the target SSAWs can reverse the particle until it reaches the right pressure node. 

On the basis of the numerical and experimental results, our method has potential applications with the created acoustic pressure field to separation of mixing particles of different sizes, gathering particles to the pressure nodes or sorting particles by switching frequency. There exists other novel particle manipulation methods, such as using phase controlled and fluorescence activated cell sorting with SSAWs and travelling SAWs (TSAWs). These methods typically use a single frequency for all the waves which are excited and having invariant pressure fields along the flow direction. Overriding the single frequency method, decaying opposing TSAW (DOTSAW) method was developed based on the ARF generated by the frequency-dependent decaying TSAW oppositely acting on the particles [63]. The method involves the use of the dual-frequency decaying SAWs excited using single-pitch IDTs and a much wider range of lateral migration than that by SSAW method of similar wavelength can be achieved. Compared with these novel methods, our method has relative merits to cooperate with different techniques, for example, the two-pitch IDT designs can be applied to the phase control method to enhance the manipulation functions. Moreover, the series IDTs create different pressure field in two regions along the flow direction. This result merits the potential application of two-stage manipulation that combines, for example, particle gathering function at the first stage and particle separating function at the second stage in one device with one operation.

## 4. Conclusions

We have investigated acoustophoretic control of particle transport in a microfluidic channel using dual-wavelength SSAWs. The dual-wavelength SAAWs composed of spatially superimposed and separated target SSAWs and non-target SSAWs were excited by parallel and series two-pitch IDTs, respectively. Numerical simulations revealed the variations of the acoustic pressure fields induced by the non-target SSAWs and their influences on particle motion. The acoustic pressure fields and particle trajectories induced by ARF were obtained to elucidate the influence of dual-wavelength SSAWs with effectively designed devices. Some design considerations arising from the existence of non-target SSAWs were proposed. The corresponding effective dual-wavelength SSAW acoustofluidic devices with two types of IDT designs were fabricated and the switching of acoustic pressure fields was experimentally achieved to transport the particles into different outlets with observation of pre-actuation by non-target SSAWs. Comparatively, the parallel IDT design is relatively suitable for multi-channel transport of the particles laterally along the channel width by generating one pressure node a time. On the other hand, the series IDTs have relative merit of the resulting pressure field that can generate two nodes a time at the two actuation regions, respectively. Therefore, the series IDT design is possible for the application of two-stage transport of particles along the channel flow direction. By combining the parallel and series IDTs, novel IDT design can be further planed for simultaneous multi-channel and multi-stage transport in the channel for advanced applications. The present study provides comprehensive information for the design of acoustofluidic devices based on multi-wavelength SSAWs that can be used for acoustophoresis.

## Figures and Tables

**Table 1:** IDT geometrical parameters.

IDT Design		Parallel	Series
Pitch (μm)	*p*_1_	398	398
	*p*_2_	363	363
Aperture (μm)	*w*_1_	2000	2000
	*w*_2_	2000	2000
Number of Pairs (*p*_1_ and *p*_2_)		20 + 20	20 + 20
Delay Length (μm)	*D*_1_ + *δ*_1_	18,010	2886
	*D*_2_ + *δ*_2_	3357	3357
Right-Side IDT Translation (μm)	*δ*_1_	25	25
	*δ*_2_	–29	–29

**Table 2:** Material parameters used in the calculations.

LiNbO_3_ [52]		Value	Unit
Elastic Constants	$c_{11}^{E}$	20.3	10^10^ N/m^2^
	$c_{12}^{E}$	5.3	
	$c_{13}^{E}$	7.5	
	$c_{33}^{E}$	24.5	
	$c_{44}^{E}$	6.0	
	$c_{14}^{E}$	0.9	
Piezoelectric Constants	*e*_15_	3.7	C/m^2^
	*e*_22_	2.5	
	*e*_31_	0.2	
	*e*_33_	1.3	
Permittivity	$\varepsilon_{11}^{S}$	38.9	10^−11^ F/m
	$\varepsilon_{33}^{S}$	25.7	
Mass Density	*ρ*_LN_	4700	kg/m^3^
PDMS [54]			
Speed of Sound	*c*_PDMS_	1000	m/s
Mass Density	*ρ*_PDMS_	970	kg/m^3^
Attenuation Coefficient	*α*_PDMS_	947	Np/m
Water [34]			
Speed of Sound	*c*_f_	1495	m/s
Mass Density	*ρ*_f_	998	kg/m^3^
Attenuation Coefficient	*α*_f_	4.22	Np/m
Dynamic Viscosity	*η*	0.893	mPa·s
Polystyrene [34]			
Speed of Sound	*c*_p_	2350	m/s
Mass Density	*ρ*_p_	1050	kg/m^3^
